# Knowledge structure and dynamic evolution of nanomedicine in liver cancer research: a scientometric analysis and visualization

**DOI:** 10.3389/fphar.2025.1509259

**Published:** 2025-02-26

**Authors:** Shaodong Li, Dapeng Cui, Bo Shao, Zhenhua Kang, Guoqiang Yan

**Affiliations:** ^1^ Department of Hepatobiliary and Pancreatic Surgery, General Surgery Center, The First Hospital of Jilin University, Changchun, China; ^2^ Department of General Surgery, The First Affiliated Hospital of Hebei North University, Zhangjiakou, China; ^3^ Department of General Surgery, Tianjin Medical University General Hospital, Tianjin, China; ^4^ Department of Colorectal and Anal Surgery, General Surgery Center, First Hospital of Jilin University, Changchun, China

**Keywords:** nanomedicine, liver cancer, bibliometric analysis, visualization, citespace, VOSviewer

## Abstract

**Background:**

Nanomedicine has received much attention for its potential applications in the diagnosis and treatment of liver cancer. However, no bibliometric evaluation has been conducted to present an assessment of scientific progress in the field. The aim of this study is to comprehensively catalog the cooperation and influence of journals, countries, institutions, and authors in the field of nanomedicine in liver cancer from the perspective of bibliometrics, evaluate the clustering evolution of knowledge structure, and uncover hot topics and emerging themes.

**Methods:**

Articles and reviews related to nanomedicine and liver cancer were retrieved from the Web of Science Core Literature Library using Topic Search. #1 T= (“Hepatic Neoplasm*” OR “Liver Neoplasm” OR “Liver Cancer*” OR “Hepatocellular Cancer*” OR “Hepatic Cancer*“), #2 T= “nano*“, the search strategy is set as #1 AND #2 limited to Science Citation Index Expanded database source, with no limitation of publication types and language/time. Bibliometric studies were conducted using CiteSpace and VOSviewer.

**Results:**

2,648 articles and reviews were included from 2000 to 2024. The number of articles regarding nanomedicine in liver cancer showed an increasing trend. Analysis of the most productive journals shows that most are specialized in nanoscience and nanotechnology, pharmacology and pharmacy, and chemistry and multidisciplinary. These publications mainly come from 8,782 institutions in 297 countries led by China and the United States of America. Shao D published the most papers among the publications, while Jemal A had the most co-citations. The macroscopical sketch and micro-representation of the whole knowledge field are realized through co-citation analysis. Hepatocellular carcinoma, targeted delivery, sorafenib nanoparticles, and others are current and developing areas of study. The keywords “nanocrystals,” “biodistribution,” and “particles” also may be the focus of new trends and future research.

**Conclusion:**

In this study, bibliometrics and visual methods were used to review the research of nanomedicine in liver cancer comprehensively. The article will help scholars to gain a better understanding of the dynamic evolution of nanomedicine applications in liver cancer and point the directions for future research.

## 1 Introduction

Hepatocellular carcinoma (HCC, also called primary liver cancer) was the sixth most commonly diagnosed cancer and the third leading cause of cancer death worldwide in 2020, with approximately 906,000 new cases and 830,000 deaths, with a 5-year survival rate of only 18% (Sung et al., 2021). Operative resection, liver grafting, local radiotherapy or chemotherapy, and combination therapy are the mainstay of treatment for HCC. However, due to the insidious onset of HCC and the lack of any specific early markers, most patients are diagnosed with HCC at an advanced stage, which is generally not amenable to operative treatment. Immunotherapy to reverse the tumor immunosuppressive microenvironment shows potential to improve antitumor therapy for patients with HCC. This involves chimeric antigen receptor (CAR) T cells, vaccines, and immune checkpoint inhibitors (ICIs) like programmed death receptor 1/programmed death receptor ligand 1 (PD-1/PD-L1) and cytotoxic T lymphocyte-associated protein 4 (CTLA-4) (Donne and Lujambio, 2023). Despite promising advances in immunotherapies (e.g., approved activating cytokines and checkpoint-blocking drugs), limited efficacy and safety are still tremendous challenges for HCC immunotherapies (Liu and Webster, 2007). Consequently, there is an urgent need for better therapeutic alternatives that inhibit tumorigenesis and/or restore sensitivity to immunotherapy-resistant tumors with liver cancer that cannot have surgery.

Nanomedicine has become a highly promising branch of nanotechnology in the 21st century (Liu and Webster, 2007). This growth is particularly significant in medical fields where traditional methods are suboptimal, such as cancer management, or treatments have been outstripped by illnesses like antibiotic-resistant bacteria (Halbus et al., 2017; Perko and Mousa, 2022). Within pharmaceutical delivery, nanomedicines are therapeutic agents formulated with polymers, lipids, or inorganic nanoparticles (NPs) with dimensions of about 100 nm or less. Several NPs have an intrinsic therapeutic effect, and nearly all types of NPs can be used as carriers for nanodrug delivery systems (NDS). These characteristics have been applied increasingly in HCC immunotherapies to improve the effectiveness and diminish the toxicity of immune-modulating drugs (Upaganlawar et al., 2022). For example, nab-paclitaxel (albumin nanoparticles of paclitaxel) and atezolizumab (PD-L1 blocker) are used to treat patients with advanced HCC. Qiu et al. reported a nanosized ultrasound contrast agent (arsenic trioxide (ATO)/PFH NPs@Au-cRGD) that integrates diagnostic and therapeutic properties for efficient ultrasound imaging and liver cancer treatment (Qiu et al., 2023). Multifunctional Janus nanocomposites with magnetic Fe_3_O_4_ heads and mesoporous SiO_2_ bodies contain doxorubicin (DOX) “nanobullets” (M-MSNs-DOX) that significantly inhibit tumor growth and reduce systemic toxicity (Shao et al., 2016). Immunotherapy with nanotechnology not only enables the precise release of medications but also further improves the therapeutic effect of antitumor agents by regulating the microenvironment (alleviating immunosuppression, reversing interstitial cell phenotypes, up-regulating or down-regulating the level of chemokines or cytokines, alleviating hypoxia, improving the level of antigen presentation, inhibiting neovascularization or improving the morphological form of existing vascularization, and tumor vaccines, etc.). Due to the great potential of nanomedicine in liver cancer, scholars have a strong interest in further understanding the role and application of nanomedicine in liver cancer, with published articles having increased rapidly. In recent years, studies on nanomedicine in liver cancer have been reviewed from various aspects. Although there is a very small amount of literature addressing this issue (Fan et al., 2024), but it is limited to nanomaterials. However, no comprehensive and impartial assessment of trends in publication, research fields, countries/regions, institutions, or authors has been undertaken in nanomedicine in liver cancer, affecting research areas and their cooperation, knowledge base, hotspots, and frontiers.

Such an exponentially visible increase in documents requires new approaches to analyse trends in the field of knowledge. Bibliometrics enables researchers to see how evidence is linked to reveal the structure and evolution of an area through two kinds of different methodologies, namely, performance analysis and bibliometric mapping, while systematic mapping offers a landscape of the current knowledge and identifies fields that need further attention and full integration (Zupic and Čater, 2015; Haddaway et al., 2016). The scientometric analysis, combining bibliometric analyses and systematic mapping, focuses on the system and features of research literature and has been applied widely to qualitative and quantitative analysis of scientific documents to understand the relationship between knowledge structure and research hotspots (Nakagawa et al., 2019). The contributions of different countries/regions, organizations, scholars, and publications can be compared to describe and predict the future progress of a specific research topic through scientometric analysis (Garcia-Fuentes et al., 2022). Many scholars have applied scientometric analysis to various fields of medicine, such as cardiovascular disease (Chen C. et al., 2020), cancer (Shen J. et al., 2022), psychopharmacology (Sabe et al., 2022), biological signaling molecule (Fang et al., 2020; Huang et al., 2021), ferroptosis (Zhang et al., 2021), and pyroptosis (Ma et al., 2021b). They are evaluating the research frontiers of nanomedicines for the treatment of liver cancer and developing treatment options. This study used CiteSpace and VOSviewer as research tools, summarized the research hotspots and frontier trends of nanomedicine in liver cancer over the past several years, and formed the corresponding knowledge map. This study will provide the current research status, clustering evolution path, frontier hotspots, and future research trends of nanomedicine in liver cancer for basic research and clinical application.

## 2 Materials and methods

### 2.1 Retrieval strategy and data collection

Several databases have unique advantages and limitations in literature retrieval, such as Web of Science (WoS), PubMed, and Scopus (Mongeon and Paul-Hus, 2015). This study used the Expanded (SCI-Expanded) of the Web of Science Core Collection (WoSCC) catalog database developed by Thomson Scientific to conduct literature retrieval, data extraction, and scientometric analysis (Leydesdorff et al., 2013). The WoSCC is considered one of the most standardized, comprehensive, consistent, and practicable scientific literature sources with the highest qualitative index (Bettencourt and Kaur, 2011). Its diversification, capacity to analyse productivity across journals, countries/regions, institutions and authors, and better compatibility with critical data analysis tools, further increase its availability. #1 TS= (“Hepatic Neoplasm*” OR “Liver Neoplasm” OR “Liver Cancer*” OR “Hepatocellular Cancer*” OR “Hepatic Cancer*“), #2 TS= “nano*“, the search strategy is set as #1 AND #2 limited to Science Citation Index Expanded database source, with no limitation of publication types and language/time. The “nano*” uses a truncated word retrieval method, which can not only save the number of characters entered, but also achieve a higher recall ratio. For example, “nano*” denotes nanomedicine, nanomaterials, nanotechnology, etc.

**FIGURE 1:**
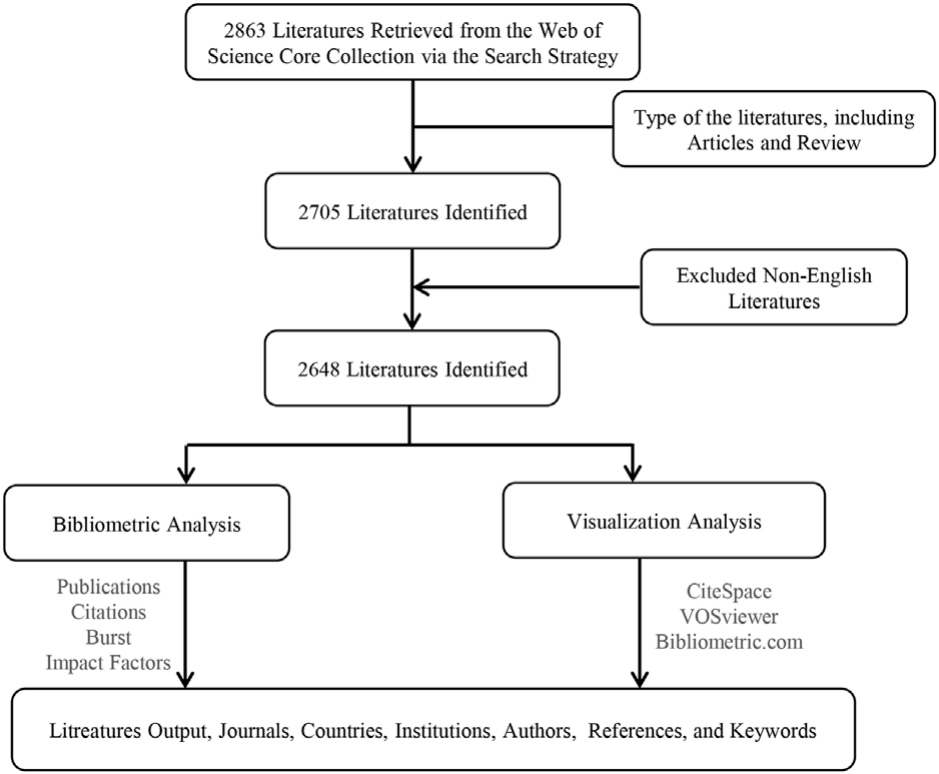
Flowchart of the filtering process.

The retrieved papers were extracted from WoSCC within 1 day (1 June 2024) to avoid bias errors in the form of “Full Record and Cited References” and saved in tag-delimited “Plain Text” files named “download_.txt.“2,863 articles were retrieved, including seven types of literature among them. We import the retrieved literature into CiteSpace for data cleaning. Only English articles and reviews are eligible for inclusion; 2,648 papers were obtained after deleting duplicate literature. The two researchers (SDL and DPC) independently searched the raw data, then discussed possible disagreements, and eventually reached an agreement of 0.96 (Landis and Koch, 1977) with considerable consistency. We finally obtained 2,648 papers; the detailed screening flowchart is shown in Figure 1.

### 2.2 Data analysis and visualization

Presently, the commonly used scientometric software includes CiteSpace, VOSViewer, Bibliometric R packages, SCI2, NetDraw, Ucinet, and HistCite (Leydesdorff et al., 2013; Mongeon and Paul-Hus, 2016). Considering their advantages and characteristics, this study used CiteSpace (version 6.3. R1) (Chen, 2004) and VOSviewer (1.6.17) (van Eck and Waltman, 2010) to conduct the data analysis and visualization.

Developed by Professor Chaomei Chen, CiteSpace is a scientometric and visual analysis tool dedicated to exploring collaborations, internal structures, hotspots, and possible trends in a field. Furthermore, CiteSpace also provides various significant metrics, including temporal metrics such as structure metrics (e.g., betweenness centrality, modularity, and silhouette), burstiness, and their combination, the sigma metric values. The specific parameters used in CiteSpace were set as follows: link retaining factor of 3.0, look back years (5), time slicing (from January 2001 to June 2024 years per slice = 1), text processing (author keywords, keywords plus, title and abstract), node type (one option chosen at a time from country, institution, author, keyword, co-cited author, and co-cited reference), links (strength: cosine, scope: within slices), pruning (Minimum et al. Sliced Networks) and others followed the default. Specifically, the g-Index is an author-level metric based on the citation frequency distribution, which inherits all the sound characteristics of the h-index, better considers the citation scores of top papers, and mitigates the bias of highly cited articles (Egghe, 2006). The magnified value of the g-Index gives credit to the low-cited or non-cited papers as similar to the high-cited papers, making the co-cited analysis clusters more comprehensive and professional. The value g-Index was adjusted to 25 for all analyses in CiteSpace. Nodes with high betweenness centrality typically connect different clusters, suggesting essential hubs in the network (in addition to closeness centrality, degree centrality, etc.) (Can et al., 2021). CiteSpace uses this indicator to find and quantify the value of literature, and a purple circle is used to emphasize such literature (or countries/regions, institutions, authors, etc.). Burstness measures the rate of variation, with the frequency of an emergent term over time indicating a specific duration when an abrupt change in the frequency takes place (Kleinberg, 2003). Using the likelihood ratio test (LLR), the cluster labels are obtained from the noun terms of the citing literature titles in the corresponding clusters (p < 0.001). Each cluster is scrutinized, and if needed, the labels formed by the automatic clustering are relabeled based on the author’s professional knowledge.

VOSviewer, developed by Leiden University, does fantastic work in creating, visualizing, and exploring maps with network-based data (Can et al., 2021). VOSviewer (version 1.6.20.0) generated scientific categories, keyword co-occurrence, and clustering maps for text-based data. We used natural linguistic algorithms to extract terms from the title and summary segments and supplemented the VOSviewer corpus files. We cleaned the data by combining “hepatocellular-carcinoma”, “liver cancer”, and “liver cancer” as “hepatocellular carcinoma” and excluding nominal terms such as “*in vitro*”, “*in vivo*”, and “roles” in keywords co-occurrence analysis.

Microsoft Office Excel 2021 was used to manage the number of articles published in the year. CiteSpace and VOSviewer software were used to analyze the distribution of journals, countries/regions, institutions, authors, and co-cited authors, as well as the dual map of journals, cluster map, and keyword co-occurrence. Besides, the latest impact factor (IF) and JCR partitions of journals were obtained from Web of Science.

## 3 Results

### 3.1 Temporal trends of publications

**FIGURE 2:**
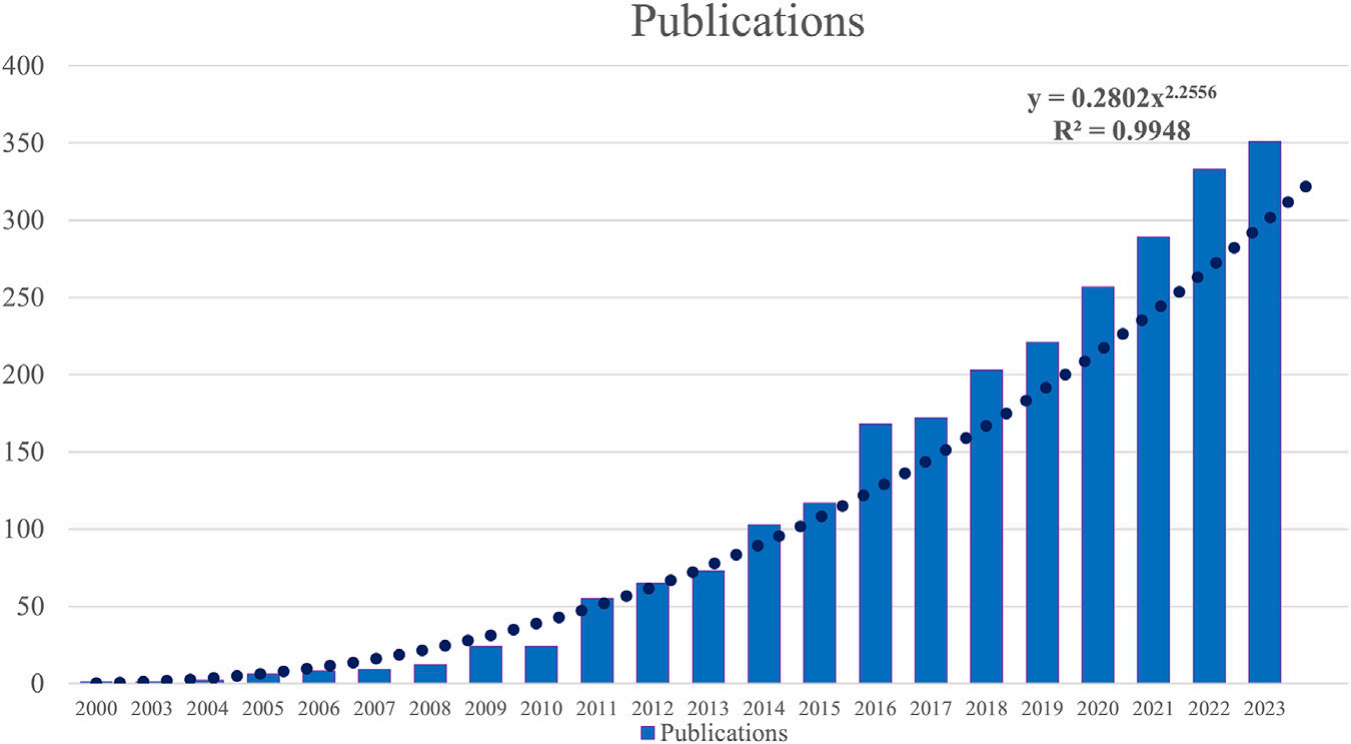
Annual publication volume of the nanomedicine in liver cancer.

The change in the number of annual publications reflects the speed and progress of this research and the degree of research emphasis on this topic (Feng et al., 2024). Figure 2 shows 2,648 articles on nanomedicine in liver cancer, showing an annual increasing trend from 2001 to 2023 (Figure 2). Between 2001 and 2010, there was a gradual and steady upward trend in the number of publications. A more significant publication increase occurred after 2011. The logistic growth curve f (x) = 0.2802*x^2.2556^ for the global publication accumulation. This area is predicted to maintain favorable development over an extended period. While the data for 2024 are incomplete, it was estimated that the eventual number of publications in 2024 will rapidly increase to over 350.

### 3.2 Scientific categories and information flow among journals

**FIGURE 3:**
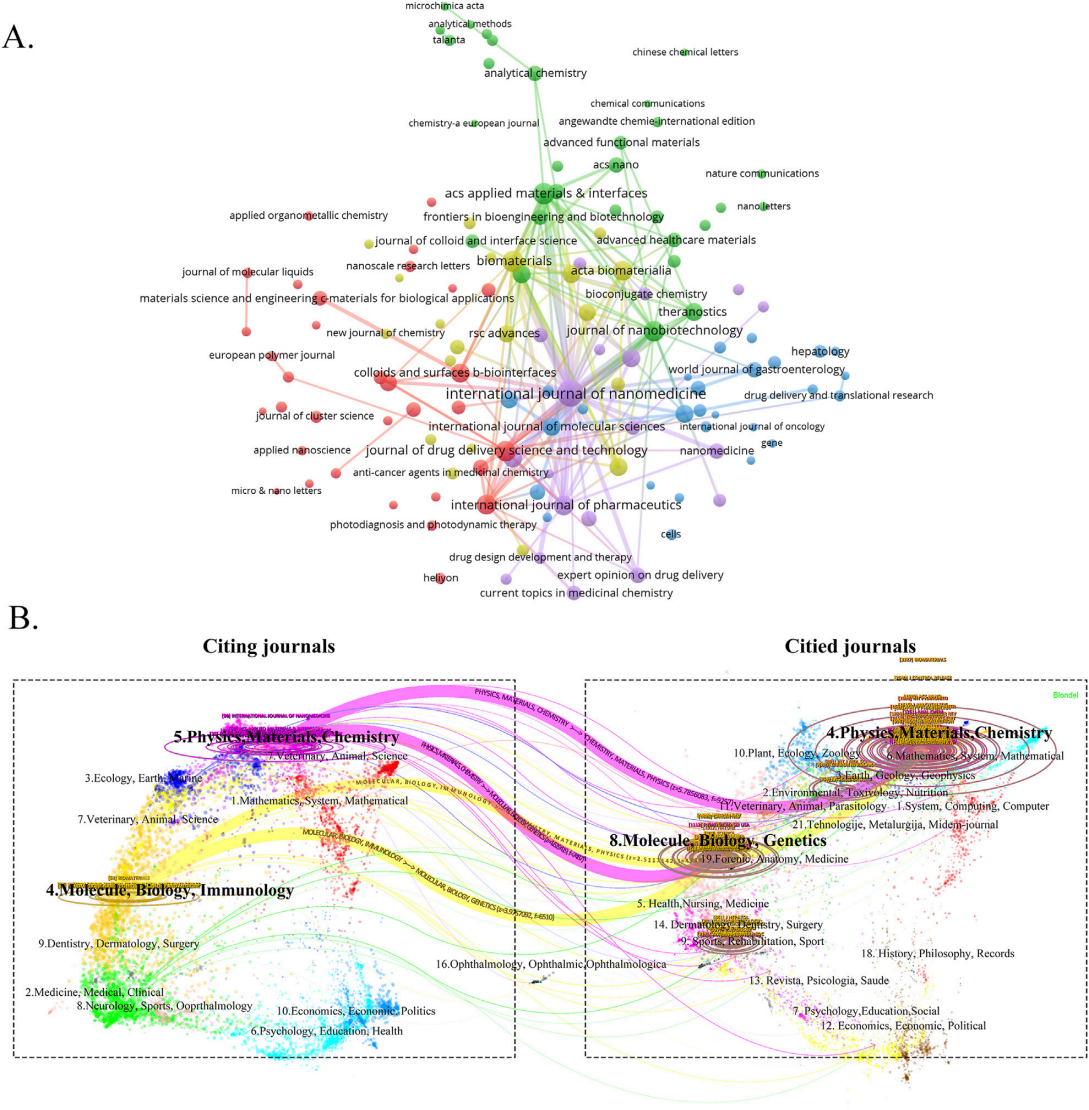
**(A)** The visualization map of journals publishing papers on nanomedicine applications in liver cancer. **(B)** The dual-map overlay of the relevant journals. Cross-flow of information between scientific categories and journals. The citing journals on the left, cited journals on the right, colored paths represent citation relationships.

In the WoSCC database, classifying journals into scientific categories provides insight into the scientific discipline and field on which the papers published in journals are concerned (Wang Y. et al., 2022). To search for the most productive and influential journals, we used VOSviewer software to visualize published journals related to nanomedicine in liver cancer (Figure 3A). The results showed that 2,648 articles were published in 702 academic journals. As shown in Table 4, the International Journal of Nanomedicine (90 publications, IF: 6.600) published the most articles concerning nanomedicine in liver cancer, followed by Biomaterials (52 publications, IF: 12.800) and ACS Applied Materials & Interfaces (75 publications, IF: 8.300). Through the analysis of the co-citation of periodicals, we can see the contribution of each periodical to this field. Among 32,196 co-cited journals, two journals had citations of over 1,000. As presented in Table 1, the Biomaterials had the most citations (citations: 1,247, IF:14.000), followed by the Journal of Controlled Release, International Journal of Nanomedicine, and ACS Nano. According to the 2023 Journal citation reports (JCR), all were at the Q1 JCR division. Five of the top ten cited journals had an IF of more than ten, and eight were from the United States.

**TABLE 1:** Document types of the publications.

Rank	Category	Count	Co-cited journal	Citation	JCR	If (2023)
1	Nanoscience & Nanotechnology	546	Biomaterials	1,247	Q1	14.0
2	Pharmacology & Pharmacy	502	Journal of Controlled Release	1,035	Q1	10.8
3	Chemistry, Multidisciplinary	395	International Journal of Nanomedicine	903	Q1	8.0
4	Materials Science, Multidisciplinary	362	ACS Nano	893	Q1	17.1
5	Materials Science, Biomaterials	313	Cancer Research	790	Q1	11.2
6	Biochemistry & Molecular Biology	217	ACS Applied Materials & Interfaces	768	Q1	9.5
7	Oncology	214	Advanced Drug Delivery Reviews	755	Q1	16.1
8	Physics, Applied	187	Journal of the American Chemical Society	745	Q1	15.0
9	Chemistry, Physical	179	International Journal of Pharmaceutics	718	Q1	5.8
10	Engineering, Biomedical	177	Proceedings of the National Academy of Sciences of the United States of America	714	Q1	11.1

To further explore the flow of knowledge between journals, especially between citing and cited journals, a dual-map overlay of the journals was developed to analyze the connection of scientific categories to nanomedicine in liver cancer, as shown (Figure 3B). This approach is designed to identify patterns of knowledge flow from cited to citing journals and provides high-level insights into innovative research output in the field of nanomedicine in liver cancer (Goerlandt et al., 2022). Designed by Chen and Leydesdorff L, the map portraying the interconnectedness of over 10,000 scientific journals is further classified into regions representing discipline-level publication and citation activity (Chen and Leydesdorff, 2014). For better interpretation, labels are presented on the dual-map overlay, which indicates clusters of journals similar to this topic. Figure 3B shows a dual-map overlay concerning nanomedicine in liver cancer articles published between 2001 and 2024. All colored curves starting from the citing (the left) map and ending at the cited (the right) map represented the paths of the citation links. Four dominant citation paths were identified. It indicated that papers published in “Physics, Materials, Chemistry” journals and “Molecular, Biology, Immunology” journals were often cited in papers published in “Molecular, Biology, Genetics” journals and “Physics, Materials, Chemistry” journals.

### 3.3 Spatial collaboration map of countries/regions and institutions

**FIGURE 4:**
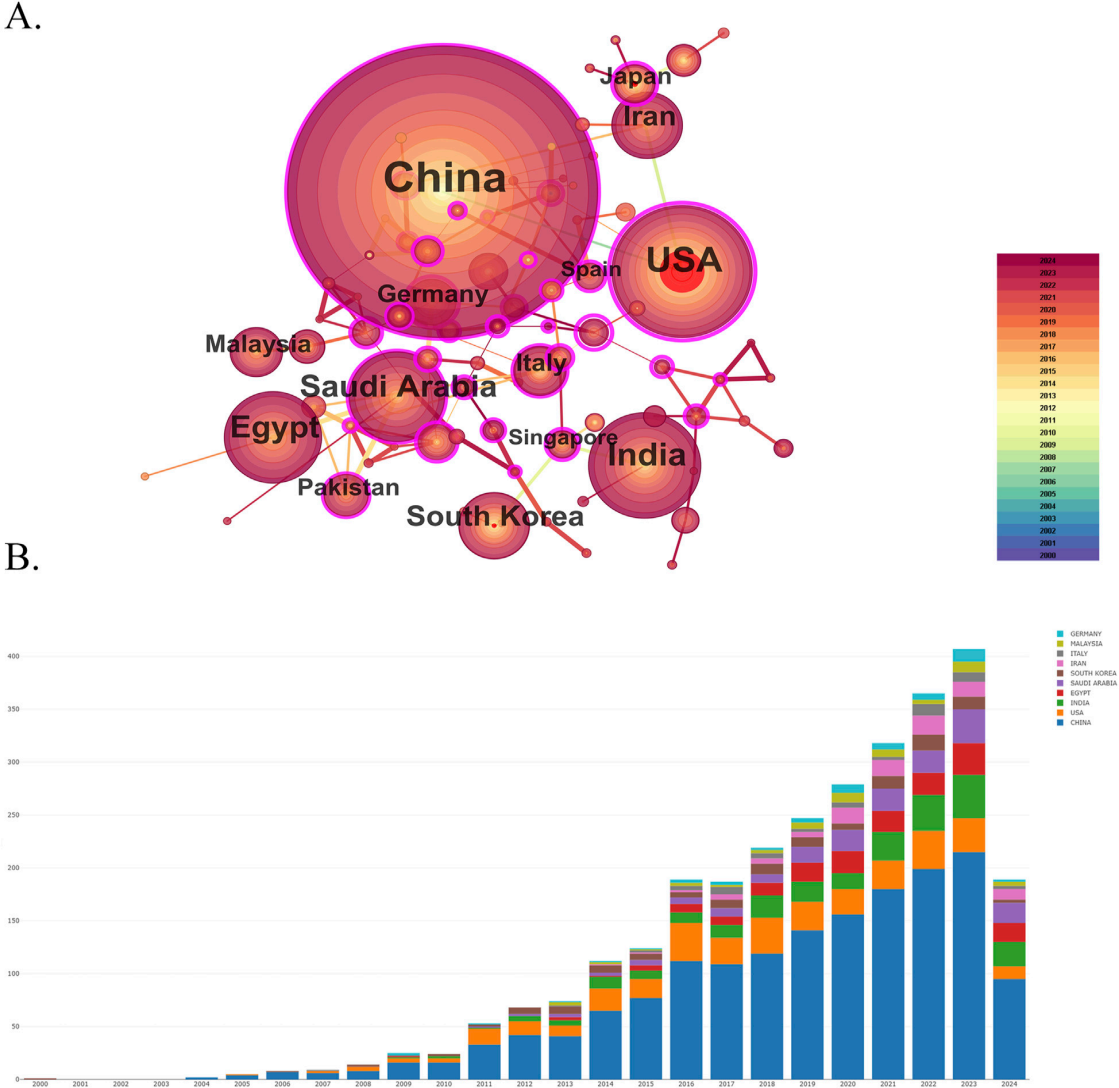
Spatial collaboration map of countries/regions. **(A)** The visualization map of leading countries contributing to research on nanomedicine applications in liver cancer. **(B)** Annual publication of leading countries contributing to research on nanomedicine applications in liver cancer.

**FIGURE 5:**
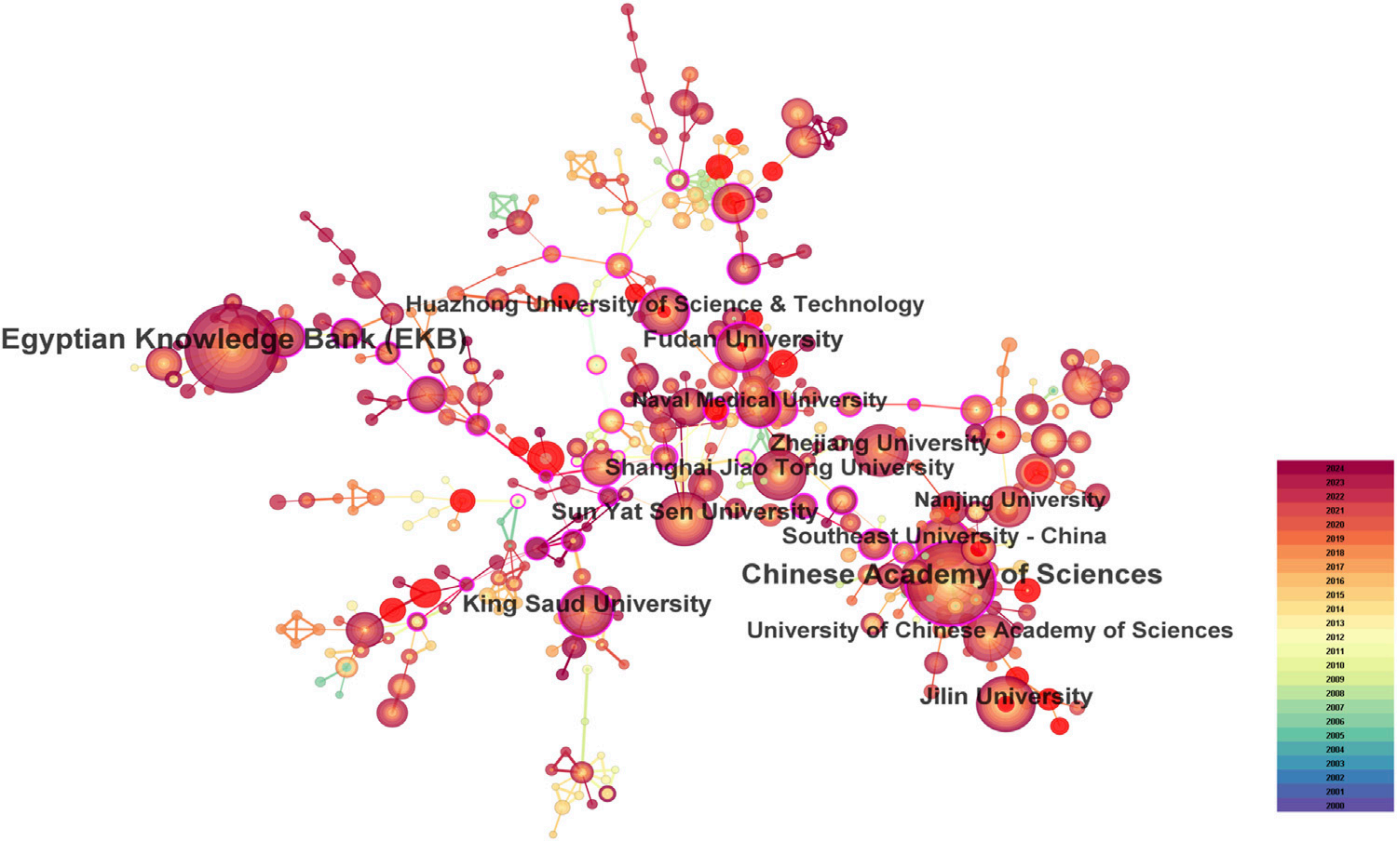
Spatial collaboration map of institutions.

This study analyzed 8,782 institutions from 297 different countries/regions contributing to the publications in the field of nanomedicine in liver cancer. As shown in Figure 4, this visualized map allowed us to identify the impact and burstness of the most critical countries/regions and institutions with significant hotspots. We ranked the ten foremost productive countries/regions and institutions according to Table 2. China (1,559/58.87%) and the USA (345/13.03%) published the most articles, which even approach seven times higher than those of other countries, followed by India (235/8.87%), Egypt (165/6.23%), and Saudi Arabia (164/6.19%). Among the top 10 productive countries, Germany (0.53), the USA (0.42), China (0.14), and Italy (0.12) were colored purple in Figure 4A with high betweenness centrality, which was generally regarded as an essential turning point, which served as a bridge structure and may lead to revolutionary discoveries. Furthermore, the Egyptian Knowledge Bank (EKB) (163, 6.16%) published the most papers, followed by the Chinese Academy of Sciences (155, 5.85%) and Fudan University (68, 2.57%). China occupied eight of the top ten fruitful institutions and was the seat of most institutions. Additionally, the Chinese Academy of Sciences (0.34), Fudan University (0.21), and King Saud University (0.17) showed high centrality, circled in purple in Figure 5. Each circle in the map represents a nation, with the size of the circle indicating the country’s number of publications. The lines connecting circles represent international collaboration, and the broader the lines, the stronger the collaboration. However, most countries and research affiliations are dispersed, and more consistent and extensive cooperation is needed.

**TABLE 2:** Publications in the 10 most productive countries/regions and institutions.

Rank	Country/Regions	Year	Count (%)	Centrality	Institutions	Year	Count (%)	Centrality
1	China	2004	1,559(58.87)	0.14	Egyptian Knowledge Bank (EKB)	2009	163(6.16)	0.04
2	United States of America	2005	345(13.03)	0.42	Chinese Academy of Sciences	2005	155(5.85)	0.34
3	India	2009	235(8.87)	0.05	Fudan University	2006	68(2.57)	0.21
4	Egypt	2009	165(6.23)	0.05	King Saud University	2012	66(2.49)	0.17
5	Saudi Arabia	2011	164(6.19)	0.14	Jilin University	2011	61(2.30)	0
6	South Korea	2000	114(4.31)	0.09	Sun Yat Sen University	2011	59(2.23)	0.1
7	Iran	2014	92(3.47)	0.05	Zhejiang University	2012	57(2.15)	0.02
8	Italy	2007	55(2.08)	0.12	Shanghai Jiao Tong University	2007	55(2.08)	0.07
9	Malaysia	2013	54(2.04)	0	Southeast University - China	2005	54(2.04)	0.1
10	Germany	2009	52(1.96)	0.53	Huazhong University of Science & Technology	2006	49(1.85)	0.1

### 3.4 Visual analysis of authors and co-cited authors

**FIGURE 6:**
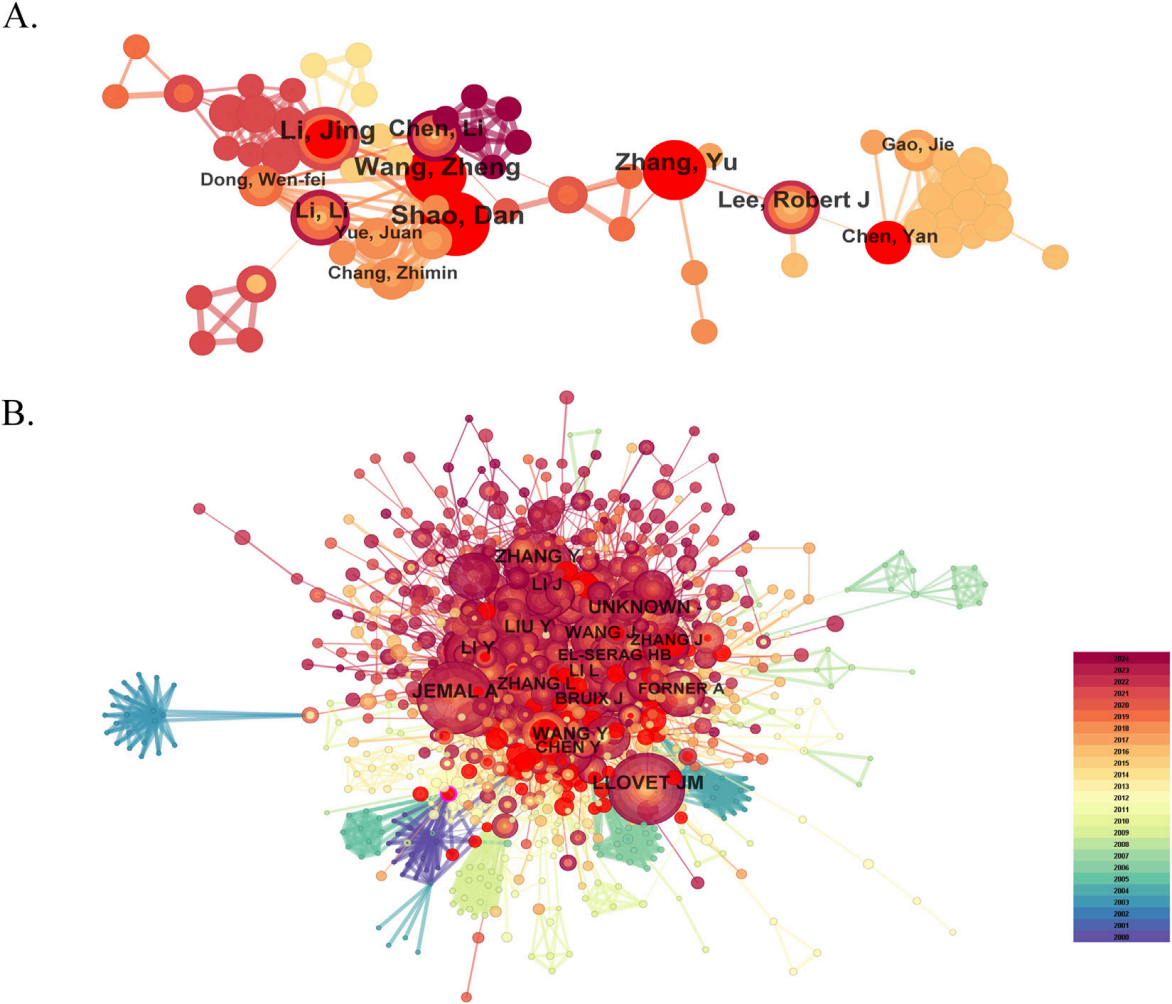
Visual analysis of authors **(A)** and co-cited authors **(B)**.

Analyzing and visualizing influential researchers, such as authors of many citing or cited papers in specific domains of science, can help researchers advance along the path and provide further opportunities and guidelines for collaboration (Ma et al., 2021a; Xue et al., 2021). There are a total of 14,773 authors and 63,989 co-cited authors associated with nanomedicine in liver cancer. The author network visualized scientific cooperation between authors using co-authors' frequency (Figure 6A). The top ten productive authors are listed in Table 3. Shao Dan of South China University of Technology for the School of Biomedical Sciences and Engineering tied for the top place in nanomedicine research toward liver cancer with the most publications published (n = 15), followed by Hussein Mohd Zobir (n = 14), Li Jing (n = 14), and Fakurazi Sharida (n = 14). Notably, the betweenness centrality was relatively slight (≤0.01), indicating that the authors had little influence on each other‘s works. The node dimension represented the number of documents published by authors, with more prominent nodal points representing more published research papers. The stronger the cooperative relationship between the two researchers, the shorter the distance between the two nodes. The purple nodes represented the authors of early published articles, while the red nodes represented the authors of recently published articles. Co-citation analysis is an essential part of scientometric analysis and visualization, and co-cited authors are referred to as two or more authors cited by another or more papers simultaneously, constituting a co-cited relationship. CiteSpace was used to analyze and visualize the co-cited author’s cooperation network regarding nanomedicine in liver cancer research. Among the top 10 co-cited authors who have been cited more than 100 times, Jemal A (271) was the most frequently cited author, followed by Llovet JM (262), Zhang Y (201), and Wang Y (196) (Figure 6B).

**TABLE 3:** Top 10 authors and co-cited authors.

Rank	Author	Count	Centrality	Co-cited author	Citations	Centrality
1	Shao, Dan	15	0	Jemal A	271	0.03
2	Hussein, Mohd Zobir	15	0	Llovet JM	262	0.02
3	Li, Jing	14	0.01	Zhang Y	201	0.03
4	Fakurazi, Sharida	14	0	Wang Y	196	0.03
5	Lin, Kuen-Song	14	0	Li Y	178	0.05
6	Wang, Zheng	13	0	Liu Y	167	0.03
7	Weng, Meng-Tzu	13	0	Wang J	159	0.04
8	Zhang, Yu	11	0.01	Li J	154	0.03
9	Chen, Li	10	0.01	Zhang L	150	0.04
10	Lee, Robert J	10	0.01	Bruix J	132	0.05

### 3.5 The knowledge base of the nanomedicine in liver cancer: co-citation and clustering network

Small and Marshakova first advanced the concept of co-citations in 1973, and it was then integrated into literature co-citation analysis to indicate that scientific literature is not an isolated resource but a system of mutual connections and continuous development (Small, 1973; Marshakova-shaikevich, 2009; Bashir, 2022). Assuming that two or more references are often cited together, it is evident that these references are associated in specific ways. It has been proved that the network map formed in this approach captures the focus of potential scientific research because the network of influential cited articles provides a knowledge basis including the main theories, concepts, and approaches, which collectively drive the generation and development of new research domain (Chen et al., 2012; Chen et al., 2014). Network clustering also provides deep insights into the degree of integration of the entire research domain since it can identify subdomains within the entire research field, which are relatively isolated from knowledge clusters based on the development of other subfields (Chen L. et al., 2020). The mapping co-citation analysis of references is the core function of CiteSpace, which characterizes knowledge structure and dynamic evolution in such networks through various visual attributes (Leydesdorff et al., 2016). Citing and cited articles represent the research frontier and knowledge base (Zhang et al., 2020). Moreover, analyzing typical clusters can help us understand nanomedicine’s knowledge structure and dynamic evolution in liver cancer. Based on the analysis of 2,648 cited articles and 106,400 valid references, the homogenous clustering of highly cited articles related to the treatment of liver cancer with nanomedicine was determined.

**FIGURE 7:**
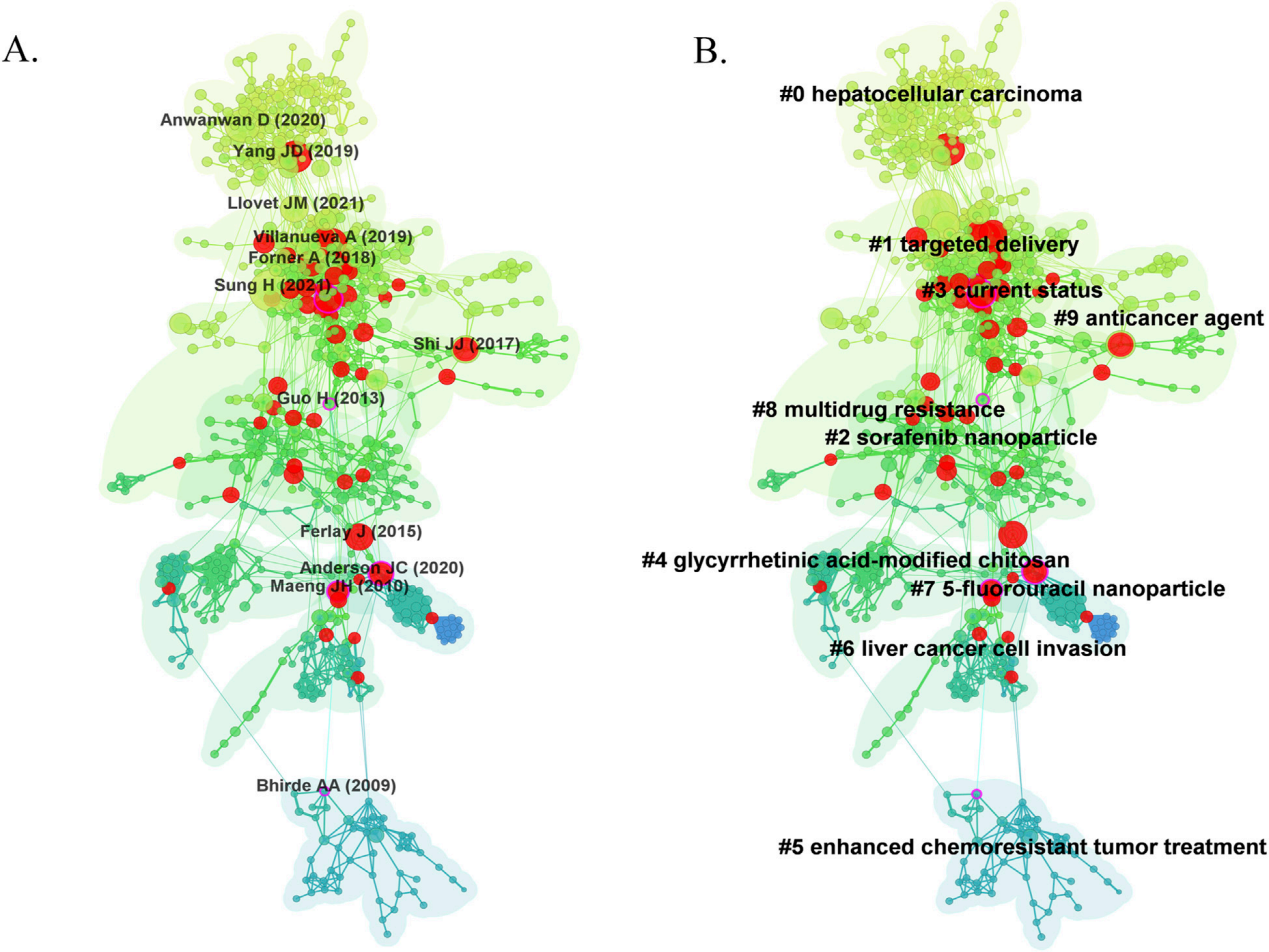
Visual analysis of co-citation **(A)** and clustering network **(B)**. The nodes in the graph represent co-cited literature and the links between the nodes represent co-citation relations. Large nodes or with red tree rings are either highly cited or exploded. All clustering labels were extracted from the titles of cited articles with a log-likelihood ratio algorithm.

The co-citation network mapping of the nanomedicine in the liver cancer research landscape was shown in Figure 7A, with the first author and the year of the top 10 most cited references. Each research article generally cited several references represented as nodes in the co-citation network map. The size of a node was proportional to the cited frequency. The links among these nodes of such reference documents represented the frequency of their references cited by the same article. Similarly, line thickness was positively correlated with co-citation frequency. More information on the top 10 cited references is presented in Table 4. The most co-cited reference performed by Sung et al. (2021) was an original article published in the Journal of Clinical Investigation, entitled “Global Cancer Statistics 2020: GLOBOCAN Estimates of Incidence and Mortality Worldwide for 36 Cancers in 185 Countries”, followed by an article entitled “Challenges in liver cancer and possible treatment approaches”. Additionally, “Functional alginate nanoparticles for efficient intracellular release of doxorubicin and hepatoma carcinoma cell targeting therapy” (0.18) showed high centrality, circled in purple in Figure 7A.

**TABLE 4:** Top 10 co-cited references.

Ranks	Title	Journal	Co-citation
1	Global Cancer Statistics 2020: GLOBOCAN Estimates of Incidence and Mortality Worldwide for 36 Cancers in 185 Countries	CA: a cancer journal for clinicians	76
2	Challenges in liver cancer and possible treatment approaches	BBA Reviews on Cancer	48
3	A global view of hepatocellular carcinoma: trends, risk, prevention and management	Nature reviews. Gastroenterology and hepatology	45
4	Cancer nanomedicine: progress, challenges and opportunities	Nature reviews. Cancer	37
5	Hepatocellular Carcinoma	The New England journal of medicine	36
6	Hepatocellular carcinoma	Nature reviews. Disease primers	35
7	Hepatocellular carcinoma	Lancet	32
8	Cancer incidence and mortality worldwide: sources, methods and major patterns in GLOBOCAN 2012	International journal of cancer	32
9	Simultaneous inhibition of growth and metastasis of hepatocellular carcinoma by co-delivery of ursolic acid and sorafenib using lactobionic acid modified and pH-sensitive chitosan-conjugated mesoporous silica nanocomplex	Biomaterials	25
10	Current status of nanomaterial-based treatment for hepatocellular carcinoma	Biomedicine and pharmacotherapy	23

Clustering network analysis can excavate the knowledge structure of the research domain (Chen, 2017). Based on the co-citation state of 106,400 references to articles cited in CiteSpace software, a hierarchical clustering network is generated if the two publications have many similar references and are often homogeneous. The most significant ten clusters extracted from the references of the 2,648 citing articles are shown in Figure 7B. Cluster labels were well-known noun phrases extracted from the title of citing articles using the logarithmic likelihood ratio (LLR) algorithm, including #0 hepatocellular carcinoma, #1 targeted delivery, #2 sorafenib nanoparticle, #3 current status, #4 glycyrrhetinic acid-modified chitosan, #5 enhanced chemoresistant tumor treatment, #6 liver cancer cell invasion, #7 5-fluorouracil nanoparticle, #8 multidrug resistance, #9 anticancer agent (Figure 7B). The number of cluster tags is inversely related to the number of articles per cluster included. A clustering network’s modularity (Q value) indicates the extent to which a network can be divided into clusters. In contrast, the silhouette (S value) is a metric of validation and interpretation of consistency within clusters (Shibata et al., 2008). As shown in Figure 7B, the total Q value was 0.8455, indicating a well-structured network, and each cluster had a weighted mean S value of 0.9191 or higher, suggesting that the cluster quality was highly credible. Moreover, the generic clusters, #0 hepatocellular carcinoma, #6 liver cancer cell invasion, and #3 current status, addressed more challenges and current status in liver cancer. At the same time, two applications focused clusters, #1 targeted delivery, #2 sorafenib nanoparticle, #4 glycyrrhetinic acid-modified chitosan, #7 5-fluorouracil nanoparticle, and #9 anticancer agent, concentrating the application of nanomedicine in liver cancer.

**FIGURE 8:**
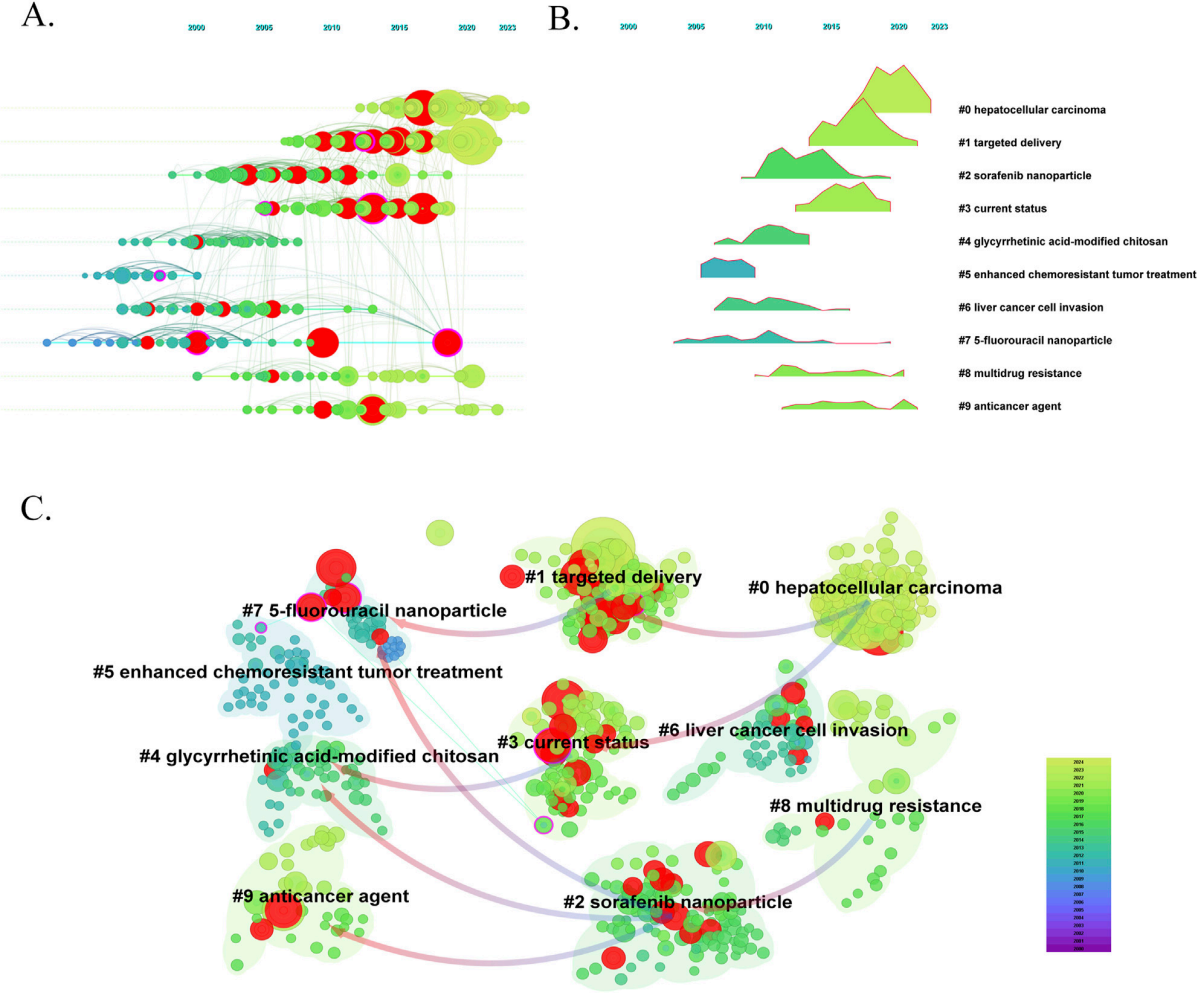
The timeline view of co-citation clusters **(A)** and **(B)**. The dependency analysis of co-cited references **(C)**.

We conducted a timeline view of co-cited references to reveal the research trends and hotspots over time (Figures 8A, B). Cluster #1 has a high concentration of nodes with citation bursts from 2014. Cluster #0 has a sustained period of about 5 years from 2006 to 2011, whereas cluster # 5 is short lived, with an associated period of 5 years from 2008 to 2015. The results showed that clusters #0 and 1 have the largest nodes scattered along the timeline and contain 4 of the 10 most frequently cited references. Besides, it is obvious that the research on 5-fluorouracil nanoparticle (cluster #7) with the size of 21 publications started earlier and relevant studies has a certain interruption period. To further explore the flow of knowledge between clustering networks, we conducted a clustering of dependency analysis of co-cited references (Figure 8C). #0 Hepatocellular carcinoma is built on #7 5-fluorouracil nanoparticle, #3 current status. Meanwhile, #1 targeted delivery, #2 sorafenib nanoparticles, #4 glycyrrhetinic acid-modified chitosan, and #9 anticancer agents provide the basis for #8 multidrug resistance. However, #5 enhanced chemoresistant tumor treatment and #6 liver cancer cell invasion are independent of the two main pathways.

### 3.6 Keywords narrative clusters and emerging trends: analysis of keyword co-occurrence

**FIGURE 9:**
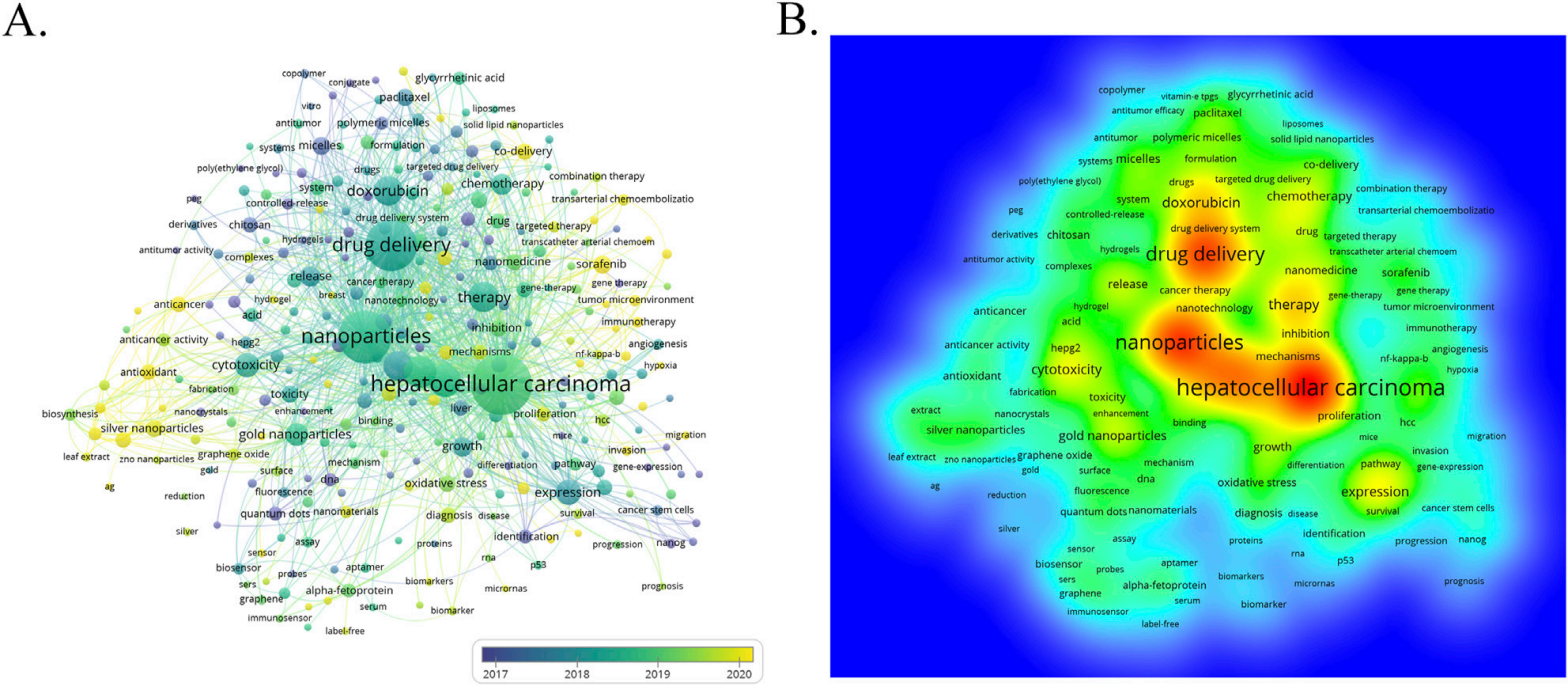
Keyword evolution over time **(A)** and keyword co-occurrence heat map analysis **(B)**. The color of each keyword indicates the average publication time of articles containing that keyword.

**FIGURE 10:**
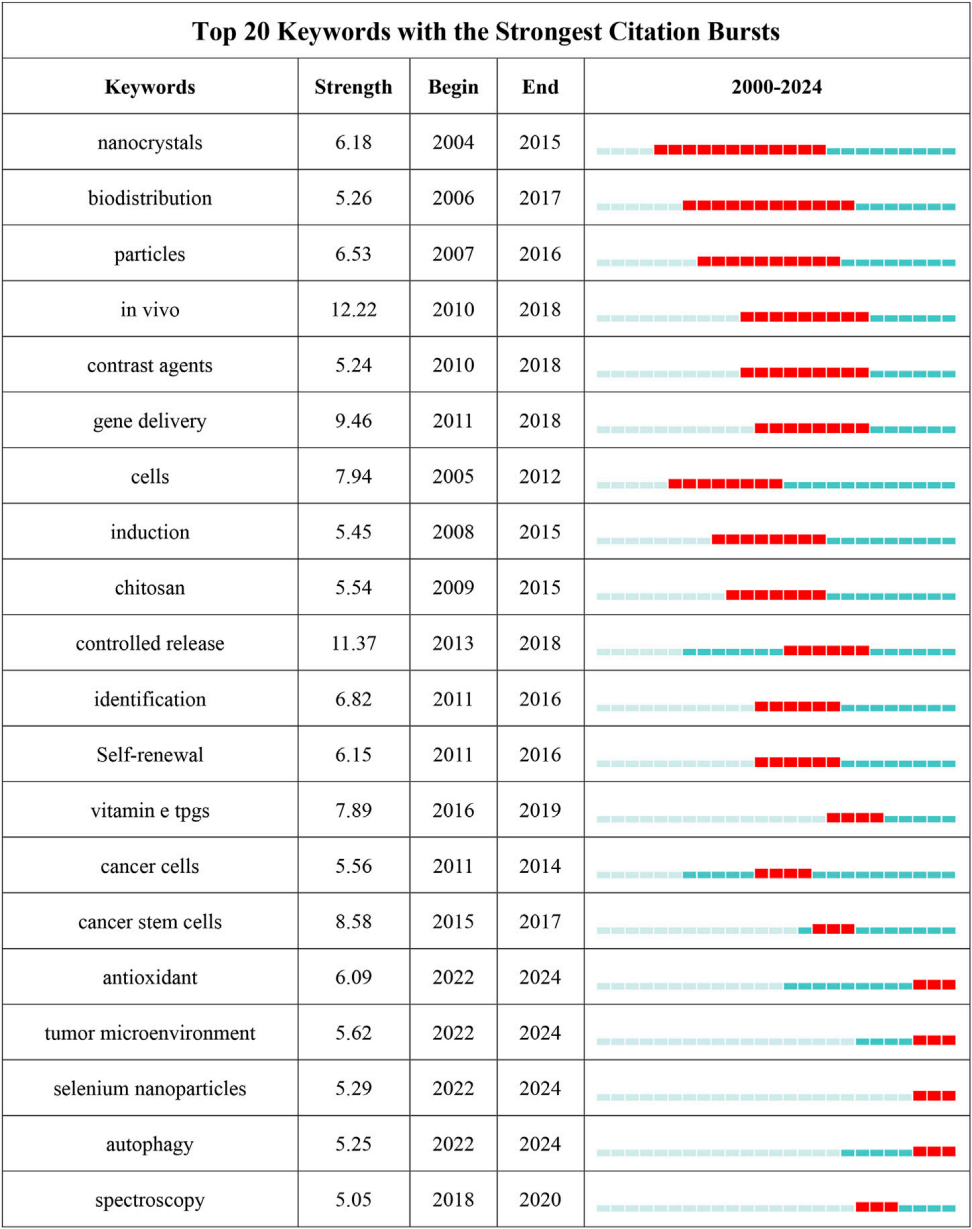
Top 20 keywords with the strongest citation bursts.

The author keywords are the core of papers, covering the main topics. In the study of scientific knowledge structure, keywords can accurately identify the research front and hotspots, which is an effective methodology of bibliometric analysis (Yu et al., 2022). The term co-occurrence (Table 5; Figure 9) and bursting keywords analysis (Figure 10) were presented using the VOSviewer and CiteSpace software, showing mutually relevant topics of nanomedicine in liver cancer. A total of 5,581 terms were extracted, among which 300 appeared more than 5 times, and 126 appeared more than 10 times. The density visualization of terms could find high frequency co-occurrence keywords, revealing research hotspots of the nanomedicine in liver cancer. As we can see from Figure 9 and Table 5, hepatocellular carcinoma is the most critical term with 1,061 co-occurrences, followed by nanoparticles, drug delivery, cancer, apoptosis, and therapy.

**TABLE 5:** The top 20 keywords.

Rank	Keyword	Occurrences	Total link strength	Rank	Keyword	Occurrences	Total link strength
1	hepatocellular carcinoma	1,061	1,045	11	gold nanoparticles	151	151
2	nanoparticles	783	771	12	chemotherapy	133	133
3	drug delivery	750	748	13	release	119	119
4	cancer	301	298	14	growth	110	109
5	apoptosis	297	296	15	micelles	96	96
6	therapy	260	259	16	sorafenib	92	92
7	cells	252	248	17	toxicity	89	89
8	doxorubicin	244	242	18	paclitaxel	86	86
9	expression	189	189	19	silver nanoparticles	83	83
10	cytotoxicity	173	170	20	iron-oxide nanoparticles	80	80

Furthermore, burst detection of keywords contributes to finding keywords that have not yet reached the frequency threshold but may have academic contributions, thereby providing a more comprehensive analysis of hot spots and fronts of the nanomedicine in liver cancer (Zhu et al., 2020). As an essential metric for researching leading-edge topics, CiteSpace detects bursting keywords (Tang et al., 2021). The timeline is represented by a line in blue, and the time span of bursting keywords is shown by the red section of the blue timeline. Figure 10 shows the top 20 keywords with the most robust citation bursts. “Nanocrystals” was the strongest burst keyword in this field from 2004 to 2015 and was followed by “biodistribution”, “particles”, and “*in vivo*”. The results suggested that studies in recent decades had increasingly focused on the relationships between the nanomaterials and antitumor drugs (e.g., chitosan, selenium nanoparticles, and Vitamin E TPGS), as well as the underlying potential applications.

## 4 Discussion

In this study, we proposed a scientometric analysis of nanomedicine’s knowledge structure and dynamic evolution in liver cancer. The WoSCC database indicated that as of 1 June 2024, 5,630 authors from 8,782 institutions in 297 countries had published 2,648 articles about nanomedicine in liver cancer. The temporal trend, scientific categories, spatial distribution, and author contributions of 2,648 retrieved articles were evaluated using CiteSpace and VOSviewer. Regarding nanomedicine in liver cancer research, we utilized literature co-citation, keyword co-occurrence, and clustering network analysis to identify the research knowledge base, hotspots, and frontiers in each period and define the topic’s evolutionary trajectory. Additionally, we identified the current research frontiers in the field of nanomedicine in liver cancer.

Feng Z et al. included a total of 1,641 literature on the research of nanomaterials in liver cancer (Fan et al., 2024), while we expanded the scope of research to the whole nanomedicine without limiting the time condition, and included a total of 2,648 literature on the research of nanomedicine in liver cancer. Therefore, we consider this study to be more comprehensive and novel. Changing annual output is a vital indicator of how a particular field is developing (Jiang et al., 2022). To address this problem, many researchers turned to the field of nanotechnology. The term was first used by Norio Taniguchi in 1974 (nanotechnology) and again by K. Eric Drexler in 1981 (nanotechnology) (Szczyglewska et al., 2023). The dramatic increase in the number of nanomedicine papers published globally in liver cancer research between 2001 and 2023. Prior to 2010, worldwide papers on nanomedicine in liver cancer research grew incrementally, but the number of these papers has increased dramatically over the past decade. According to the logistic growth model, the global turning point will likely occur in 2025. It has brought nanomedicine to the forefront and has attracted participation and support from research organizations in various countries. Consequently, there has been a progressive increase in research on the relationship between nanomedicines and liver cancer.

The overview of nanomedicine in the liver cancer research domain was initiated by analyzing the contributions of diverse scientific fields and journals. Nanoscience & Nanotechnology was the most prominent category in the field of the nanomedicine in liver cancer. Having the second highest frequency and centrality, the cross-domain of Nanoscience & Nanotechnology was highly comprehensive, indicating a revolution in multidisciplinary research on the nanomedicine in liver cancer. However, Biotechnology & Applied Microbiology, Electrochemistry, Biophysics, and Radiology, Nuclear Medicine & Medical Imaging were also breakthrough points with relatively high centrality. Additionally, the dual-map overlay of journals suggested a strong predominant pattern in knowledge flow between publications and that most of the literature on nanomedicine in liver cancer was published in a relatively limited set of journals (Shen Z. et al., 2022; Wang C. et al., 2022). Four major citation trajectories, indicating that nanomedicine-related research in liver cancer favors translational medicine and cross-disciplinary-oriented categories. Journal and cited journal analyses can provide a significant amount of information, which can assist researchers in choosing appropriate journals for submission. Our study showed that nearly two-fifths of the papers published in the top ten most active journals covered the topic of nanomedicine in liver cancer, indicating that the distribution of the literature is relatively concentrated. International Journal of Nanomedicine (90 publications, IF: 6.60) published the most papers, while Biomaterials received the most significant number of citations. Both were journals concerning nanomedicine, consistent with the dual-map overlay of journals. Additionally, it can be concluded that the study of the clinical translation of nanomedicine in liver cancer is a current hot topic and a direction for future research.

Regarding the geographic distribution of countries, regions, and institutions, China, Unite States, and India were among the top 3 most productive countries. South Korea was the first country to carry out nanomedicine studies in liver cancer, followed by China, the United States, Italy, Germany, and India; these countries were among the ten countries with the highest production. In a network structure, the betweenness centrality is typically applied to measure the significance of the bridge function of nodes (Iacobucci et al., 2018). Germany and the United States had the highest betweenness centrality, which represented one of the critical bridges in national cooperation networks worldwide. Interestingly, China ranked first in the literature published. However, the betweenness centrality was just 0.14, indicating that although the number of publications in China has grown, high-quality articles still need to be available. China accounted for about 60% of those institutions in the top ten. The Chinese Academy of Sciences, the Fudan University, and the Jilin University published the most, and we found considerable collaboration and significant betweenness centrality among these institutions, indicating their substantial contribution to nanomedicine in liver cancer-related research.

Emphasizing the influential authors through a comprehensive analysis of the authors of many citing or cited papers in a specific domain can help researchers identify potential collaborators and provide further guidance and direction (Huang et al., 2024; Yang et al., 2024). Shao Dan (15 papers) published the most papers, while Jemal A (times cited 271) had the most co-citations. Additionally, we identified two scholars, Li Jing, and Zhang Yu, who were not only the top 10 prolific authors but were also the top 10 co-cited authors, which implied that all three authors had made significant contributions in the field of the nanomedicine in liver cancer. In 2016, Dan Shao and his colleagues designed multifunctional Janus nanocomposites, which are characterized by magnetic Fe_3_O_4_ in the head and mesoporous SiO_2_ in the body, and contain doxorubicin (DOX) as a “nanobullet” (M-MSNs-DOX). It is found that under the effect of the magnetic field, M-MSNs-DOX can selectively inhibit the growth of cancer cells instead of inhibiting the growth of normal cells in the human body, utilizing nanotechnology to treat liver cancer safely and efficiently (Shao et al., 2016). Hussein Mohd Zobir has contributed significantly to the development of therapeutics and theranostic nanodrug delivery systems for liver cancer (Ruman et al., 2020). The nanocarrier-based drug delivery systems (NDDS) may provide a novel treatment approach to deliver drugs selectively to cancerous regions and reduce the chance of nonspecific delivery to healthy tissues, thereby reducing drug side effects (Zhang et al., 2019). Tang, et al identified that compared with free sorafenib (SFB), the SFB-loaded biodegradable d-tocopherol polyethylene glycol 1,000 succinate polycaprolactone nanoparticles (TPGS-b-PCL NPs) were more efficiently inhibited the growth of HepG2 cells and retarded tumor growth in HCC xenograft models (Yang T. et al., 2019). It is expected that these pioneering academic scientists will continue to have an impact on the future development of nanomedicine in liver cancer and encourage young researchers to enter them in the clinical translation of nanomedicine in liver cancer.

Co-citation analysis is a methodology that reveals intrinsic patterns that are considered to be the basis of research in a particular field (Yang L. et al., 2019). This suggests that scientists are paying close attention to this literature, which may highlight the evolving changes and emerging trends in nanomedicine in liver cancer research. The co-citation analysis for the nanomedicine in liver cancer was mainly focused on ten research areas in this case: “hepatocellular carcinoma”, “targeted delivery”, “sorafenib nanoparticles”, “current status”, “glycyrrhetinic acid-modified chitosan”, “enhanced chemoresistant tumor treatment”, “liver cancer cell invasion”, “5-fluorouracil nanoparticle”, “multidrug resistance”, and “anticancer agents”. Early co-citation clustering in the literature focused on the nanomaterials research, e.g., #2 sorafenib nanoparticle, #4 glycyrrhetinic acid-modified chitosan, and #7 5-fluorouracil nanoparticle. Caputo TM et al. developed a biodegradable and biocompatible amino-polymeric Poly (D, L-Lactide-co-glycolide) (PLGA) nanoparticle loaded with sorafenib by an emulsion-solvent evaporation process. Their high stability confirmed the enhanced cytotoxic effect of the nanoparticles on liver cancer cells, sustained release properties and rapid cellular uptake (Caputo et al., 2023). Chitosan nanoparticles are biodegradable cationic polysaccharide polymers synthesized by partial deacetylation of chitin (Hong et al., 2017). Many studies have focused on chitosan nanoparticles applied to liver cancer drug delivery for therapeutic purposes. Cheng et al, evaluated chitosan-coated 5-fluorouracil nanocarriers in hepatocellular carcinoma where it had promising inhibitory effects on liver cancer (Cheng et al., 2013). Loutfy et al. also synthesized chitosan nanoparticles for *in vitro* human hepatocellular carcinoma cell model evaluation (Loutfy et al., 2016). They found that chitosan nanoparticles had better cytotoxic effects on liver cancer cells and suggested the chitosan nanoparticles are suitable as a drug delivery option for liver cancer. Besides, other nanoparticles such as micelles (Hanafy et al., 2018), liposome (Quagliariello et al., 2019), dendrimer (Noriega-Luna et al., 2014), graphene oxide-based nanocarriers (Pan et al., 2016), polylactic-co-glycolic acid (PLGA) nanoparticles (Danhier et al., 2012), carbon nanotubes (Ravi Kiran et al., 2020), superparamagnetic iron-oxide nanoparticles (Depalo et al., 2017), are also used in the diagnosis and treatment of liver cancer.

Co-citation determined the knowledge base and clustering evolution, keyword co-occurrence analysis revealed the research frontiers and hotspots; keyword clustering analysis showed the knowledge structure, and the timeline view visualized the hotspot evolution of the keywords. All these keywords in VOSviewer are labeled with a different color according to the average year of publication. The earlier keywords are represented by blue, while the later keywords are represented by yellow. The keywords of “hepatocellular carcinoma”, “nanoparticles”, “drug delivery” and “therapy” are the main topics in the early stage. The keywords of “silver nanoparticles”, “antioxidant”, “sorafenib” and “mechanisms” exhibit relatively recent average publication years, demonstrating that this topic is potentially of great interest and concern to clinicians and scientists.

Despite encouraging results in preclinical and clinical trials, the successful clinical translation of nanomedicine for liver cancer treatment still faces several obstacles: (1) The behavior of nanomaterials is not fully understood *in vivo*, which seriously hinders the design and optimization of nanomedicines. Further study of the behavior of nanomedicines will help to better evaluate and further improve the bioavailability, biocompatibility and pharmacokinetics of nanomedicines, thereby improving anticancer efficacy and development efficiency; (2) At present, the loading efficiency of most nanomedicines is moderate, which greatly limits the efficacy of nanomedicines. This challenge can be solved by improving existing nanocarriers or preparing new nanocarriers; (3) The performance of targeted localization of nanomaterials is far from satisfactory and needs further improvement. This problem can be solved by developing new ligands for known targets or finding new targets in liver cancer biomarkers; (4) The characterization and evaluation of nanomedicine has always been a test, which requires a revolutionary means to replace the current evaluation; (5) In the promotion of nanomedicine, industrial manufacturing is still a difficult task. The large-scale production of nanomedicines remains very difficult and requires the collaborative efforts of nanotechnology experts, engineers, oncologists and chemists to overcome this challenge by developing new technologies; Despite these challenges, we believe that these problems will be adequately addressed in the future through multidisciplinary collaboration of medicine, materials science, biology, physics, chemistry, as well as engineering. A new era is awaiting us in which nanomedicine will become an important modality for liver cancer treatment.

The bibliometric analysis and visualization provide some insight into the structural and temporal dynamics of the field, but this study still has some limitations. The raw data was retrieved from the WoSCC database only, and may miss some relevant records published in other databases (e.g., Google Scholar or PubMed). However, WoSCC data represent to some extent the majority of studies and provide comprehensive and detailed information for references (Campbell et al., 1988; Fang et al., 2023). Also, WoSCC is the most widely utilized database for bibliometric analysis (Ling et al., 2023). In addition, given the limitations of literature types, terms, and languages, our retrieval strategy might not identify all relevant references; therefore, our findings needed to be more comprehensive. However, our study has thoroughly analyzed the current status of the nanomedicine in liver cancer research and its progress between 2000 and 2024, contributing to development of future research directions.

## 5 Conclusion

The application of nanomedicines in the diagnostic and therapeutic process of liver cancer has become a focus of attention. The bibliometric analysis presents an objectively quantifiable method for understanding the roles and molecular mechanisms and evaluating trends and frontiers of the nanomedicine in liver cancer. Publications in this field have increased rapidly since 2011, and there is strong international scientific cooperation, but more collaborations may be necessary among researchers. Many articles have been published in international core journals, showing great prominence. Nanoscience & Nanotechnology, Pharmacology & Pharmacy, and Chemistry & Multidisciplinary will highlight future research. This study could provide important insights for researchers into the structural and dynamic evolution of nanomedicine in liver cancer.

## Data Availability

The original contributions presented in the study are included in the article/supplementary material, further inquiries can be directed to the corresponding author.
